# Relationship between Nutrition Knowledge and Physical Fitness in Semiprofessional Soccer Players

**DOI:** 10.1155/2014/180353

**Published:** 2014-07-21

**Authors:** P. T. Nikolaidis, E. Theodoropoulou

**Affiliations:** ^1^Department of Physical and Cultural Education, Hellenic Army Academy, 16672 Athens, Greece; ^2^Exercise Physiology Laboratory, 18450 Nikaia, Greece; ^3^Faculty of Physical Education and Sport, University of Athens, 17237 Athens, Greece

## Abstract

Whereas nutrition has a crucial role on sport performance, it is not clear to what extent nutrition knowledge is associated with physical fitness. The aim of this study was to examine the current level of nutrition knowledge of soccer players and whether this level is associated with physical fitness. Soccer players (*n* = 185, aged 21.3 ± 4.9 yr, weight 72.3 ± 8.4 kg, and height 177.5 ± 6.4 cm) performed a battery of physical fitness tests (sit-and-reach test, SAR; physical working capacity in heart rate 170, PWC170; and Wingate anaerobic test, WAnT) and completed an 11-item nutrition knowledge questionnaire (NKQ). Low to moderate Pearson correlations (0.15 < *r* < 0.34, *p* < 0.05) of NKQ with age, weight, height, fat free mass (FFM), SAR, peak power, and mean power of WAnT were observed. Soccer players with high score in NKQ were older (4.4 yr (2.2; 6.6), mean difference (95% confidence intervals)) and heavier (4.5 kg (0.6; 8.3)) with higher FFM (4.0 kg (1.1; 6.8)) and peak power (59 W (2; 116)) than their counterparts with low score. The moderate score in the NKQ suggests that soccer players should be targeted for nutrition education. Although the association between NKQ and physical fitness was low to moderate, there were indications that better nutrition knowledge might result in higher physical fitness and, consequently, soccer performance.

## 1. Introduction

Soccer is a sport taxing both aerobic and anaerobic energy transfer systems. An estimate of the energy cost of training or match-play in elite players is above 1500 kcal [1]. The metabolism during the game relies on muscle glycogen and free-fatty acids [2]. Thus, soccer players should adopt a diet providing sufficient carbohydrates and supplying all nutrient requirements [3]. The intake of carbohydrates might range from 5 to 7 g per kg during moderate training to 10 g per kg during intense training or match-play [1]. The diet should include 55–65% carbohydrate, 12–15% protein, and less than 30% fat [4, 5] and should be according to soccer players' age [6]. Emphasis should also be given to have sufficient water and electrolyte levels [7].

Whereas an optimal diet is necessary to meet the abovementioned energy requirements of soccer, the existing research reveals many nutrition concerns [8–11]. For instance, in an analysis of nutrition of semiprofessional soccer players, insufficient amount of carbohydrates consumption was noticed [8]. Moreover, Garrido and colleagues compared two menu settings (“buffet-style” versus fixed “menu”) and concluded that these settings did not meet the current recommendations [9]. Ruiz and colleagues examined the contribution of carbohydrates to total energy intake in soccer players of various age groups and observed that it was lower than what was recommended for athletes [10]. In another study [11], where the nutrition of adolescent soccer players was analyzed, their total energy intake was insufficient and the diet was unbalanced with great emphasis upon fatty foods. These studies highlighted the need for an optimal nutrition.

Nutrition has a crucial role on sport performance, but it is not clear to what extent nutrition knowledge is associated with physical fitness. It is reasonable to assume that better nutrition knowledge might result in better nutrition choices, which in turn might enhance various physical fitness components (e.g., body composition, anaerobic power, and endurance). Therefore, the aim of this study was to examine (a) the current level of nutrition knowledge of soccer players and (b) whether this level is associated with physical fitness.

## 2. Methods

### 2.1. Study Design and Participants

For the purpose of this study, we collected data from 185 semiprofessional soccer players (*n* = 185, aged 21.3 ± 4.9 yr, weight 72.3 ± 8.4 kg, and height 177.5 ± 6.4 cm), who were examined in our laboratory in the beginning of preparatory period of seasons 2011-2012 and 2013-2014. The study was approved by the local review board. All participants gave written informed consent and underwent a series of anthropometric, body composition, and physical fitness measurements (sit-and-reach test, SAR; physical working capacity in heart rate 170, PWC_170_; and Wingate anaerobic test, WAnT), and completed an 11-item nutrition knowledge questionnaire (NKQ) [12].

### 2.2. Protocols and Equipment

*(a) Anthropometry*. Weight was measured with an electronic weight scale (HD-351 Tanita, Illinois, USA) in the nearest 0.1 kg and height with a portable stadiometer (SECA, Leicester, UK) in the nearest 1 mm with participants being barefoot and in minimal clothing. Body mass index was calculated as the quotient of body mass (kg) to height squared (m^2^). A caliper (Harpenden, West Sussex, UK) measured skinfolds (0.5 mm) and body fat percentage (BF) was calculated from the sum of 10 skinfolds [13]. Fat free mass (FFM) was calculated as the difference between weight and the product of weight by BF.

*(b) Flexibility*. The sit-and-reach (SAR) protocol [14] was employed for the assessment of lower back and hamstring flexibility.

*(c) Physical Working Capacity in Heart Rate 170 min* ^*−1*^ *(PWC*_*170*_). PWC_170_ was performed according to Eurofit guidelines [15] on a cycle ergometer (828 Ergomedic, Monark, Varberg, Sweden). Seat height was adjusted to each participant's satisfaction, and toe clips with straps were used to prevent the feet from slipping off the pedals. Participants were instructed before the tests that they should pedal with steady cadence 60 revolutions per minute, which was given by both visual (ergometer's screen showing pedaling cadence) and audio means (metronome set at 60 beats per minute). This test consisted of three stages, each lasting 3 min, against incremental braking force in order to elicit HR between 120 and 170 beats per minute (min^−1^). Based on the linear relationship between HR and power output, PWC_170_ was calculated as the power corresponding to HR 170 min^−1^ and expressed as W and W*·*kg^−1^. HR was recorded continuously during all testing procedures by Team2 Pro (Polar Electro Oy, Kempele, Finland).

*(d) Wingate Anaerobic Test (WAnT)*. The WAnT was performed on a cycle ergometer (Ergomedics 874, Varberg, Monark, Sweden). Briefly, participants were asked to pedal as fast as possible for 30 s against a braking force that was determined by the product of body mass in kg by 0.075 [16]. Peak power (*P*_peak_) was estimated as the average power over a 5 s period with the highest performance, which occurs usually in the first 5 s of the test. Mean power (*P*_mean_) was calculated as the average power during the 30 s period. Both *P*_peak_ and *P*_mean_ were expressed as W and W*·*kg^−1^. Fatigue index was calculated.

### 2.3. Statistical Analysis

Statistical analyses were performed using IBM SPSS v.20.0 (SPSS, Chicago, USA). Data were expressed as mean and standard deviations of the mean (SD) and parametric statistics were used, because significance value of Kolmogorov-Smirnov test of normality was lower than 0.001 for all variables. Pearson correlation coefficient *r* was used to examine the relationship between NKQ and physical fitness variables. Magnitude of correlation coefficients were considered as trivial (*r* < 0.1), small (0.1 ≤ *r* < 0.3), moderate (0.3 ≤ *r* < 0.5), large (0.5 ≤ *r* < 0.7), very large (0.7 ≤ *r* < 0.9), nearly perfect (0.9 ≤ *r* < 1.0), and perfect (*r* = 1.0) [17]. Based on their NKQ scores, participants were classified into three groups: low (less than 5 correct answers out of 11), moderate (5 or 6 correct answers), and high nutrition knowledge (more than 6 correct answers). One-way analysis of variance (ANOVA) with a subsequent Bonferroni post-hoc test (if difference between the groups was revealed) was used to examine differences in physical and physiological characteristics among the three groups. Mean difference together with 95% confidence intervals (CI) was calculated when the post-hoc was necessary. To interpret the effect size for statistical differences in the ANOVA we used eta square classified as small (0.01 < *η* ^2^ ≤ 0.06), medium (0.06 < *η* ^2^ ≤ 0.14), and large (*η* ^2^ > 0.14) [17]. The level of significance was set at *α* = 0.05. A stepwise linear regression analysis was conducted to predict the overall NKQ score from anthropometric and physical fitness components.

## 3. Results

The answers to the NKQ can be seen in Table 1. Mean score and standard deviation of NKQ (i.e., number of correct responses) were 5.4 and 1.7. Low to moderate correlations (0.15 < *r* < 0.34,   *p* < 0.05) of NKQ with age, weight, height, fat free mass (FFM), SAR, peak power, and mean power of WAnT were observed (Table 2). Soccer players with high score in NKQ were older (4.4 yr (2.2; 6.6), mean difference (95% confidence intervals)), and heavier (4.5 kg (0.6; 8.3)) with higher FFM (4.0 kg (1.1; 6.8)) and peak power (59 W (2; 116)) than their counterparts with low score (Table 3). NKQ score could be predicted from age by the following formula: NKQ = 3.0 + 0.1 × age, *R* ^2^ = 0.11.

With regards to questions on macronutrients, the majority of soccer players agreed correctly that “carbohydrates and fat are the main energy sources” and disagreed correctly that they “should consume high-fat meals 2 to 3 hours before an event” and that “eating carbohydrates makes you fat.” The number of soccer players who answered correctly to the question about the necessity of protein supplements was equal with those who answered mistakenly. The majority agreed mistakenly with the sentence that “should not eat sweets prior to an event” and that “protein is the main energy source for the muscle.”

Compared with their knowledge on macronutrients, soccer players had better performance in the questions concerning hydration. Particularly, they answered correctly three out of four questions; they agreed correctly that “should replace fluids before, during and after an event” and that “dehydration decreases performance,” and disagreed correctly with the sentence that they “should rely on thirst to ensure fluid replacements.” In contrast, they disagreed incorrectly with that “sports drinks are better than water”.

## 4. Discussion

The main findings were that the overall nutrition knowledge score of the large sample of soccer players participating in this study was evaluated as poor and this score was related to age, weight, height, fat free mass, flexibility, and anaerobic power. The comparison between groups differing in nutrition knowledge revealed differences with regards to body composition and anaerobic power. Particularly, we observed that soccer players with high score in NKQ were heavier (~4.5 kg) than their counterparts with low score. This difference reflected mainly the higher amount of FFM (~4 kg) indicating that nutrition knowledge might help soccer players increase their FFM.

The excess of FFM in the group of high score in NKQ explains partially their increased *P*_peak_ (~59 W) in the WAnT; we observed difference in *P*_peak_ expressed as W, but when the values were adjusted for body mass (i.e., W*·*kg^−1^) there was no significant difference. An interpretation of these findings might be that good nutrition knowledge results in good dietary choices, which in turn contributed to increased FFM. Thus, considering the close relationship between FFM and muscle power [18, 19], it was not surprising to observe high muscle power (in absolute values) in soccer players with high FFM.

However, these findings did not provide insights for the exact mechanisms of this relationship, because nutrition behavior was not measured. A review of studies assessing the relationship between knowledge and dietary intake supported that 5 of 9 studies reported a positive but of moderate magnitude association between these parameters [20]. This conclusion was also confirmed by a more recent review [21]. In addition to cross-sectional studies, the relationship between nutrition knowledge and behavior has been also examined with longitudinal design. Even in the latter case, the findings of previous studies were not always consistent; however, most of them indicated changes in both nutrition knowledge and behavior in the same direction. For instance, NCAA female volleyball players after one year [22] and college female athletes after an 8-week intervention improved both [23], whereas six sessions improved knowledge but not dietary intake in university elite athletes [24].

This is not the first study to observe poor nutrition knowledge in a sport population. Previous research on athletes [25–27] and nonathletes [28] had already shown important nutrition concerns. For instance, poor knowledge of the foods required for refueling, sport drinks, and the role of protein in muscle formation was found in Irish rugby players aged 15–18 yr [27]. In a research on female collegiate swimmers there was lack of knowledge of nutrition [25], whereas in a research on female collegiate cross-country runners it was suggested that these athletes lacked nutrition knowledge critical to preventing nutrition-related health problems [26]. A modest nutrition knowledge and misconception with respect to the sugar content in food or in beverages were recorded in a large sample of adolescents aged 12.5–17.5 yr [28]. Consequently, the findings of this study combined with those of previous research indicated the need for nutrition education of athletes. As the stepwise regression analysis in the present study showed, age was the best predictor of nutrition knowledge (i.e., the older the soccer player, the higher the NKQ score). The role of age was also mentioned by a study on elite Australian athletes [29]. Therefore, educational interventions regarding nutrition should target younger players.

A limitation of this study was that this questionnaire has not been previously validated against other measures of nutrition knowledge. However, the questionnaire has face validity [30], because it covered most of the content it was supposed to measure (e.g., carbohydrates and fats as energy sources and role of proteins, water, minerals, and vitamins) [31]. On the other hand, despite the advancement of research in the field of nutrition since the development of this questionnaire in 2002, our findings revealed its content to be quite timely (e.g., the misconceptions that “protein is the main energy source for the muscle” and “vitamin and mineral supplements increase energy levels”). Moreover, this is the first study to examine the relationship between nutrition knowledge and physical fitness in soccer players and, thus, our findings can be used as a reference for future research on soccer players' nutrition knowledge.

## 5. Conclusions

The moderate score in the NKQ suggests that soccer players should be targeted for nutrition education. Based on our findings, a nutrition intervention should aim to educate soccer players especially with regard to the role of proteins and vitamins, both of which were considered mistakenly by the majority of subjects to increase energy levels. Even if the association between NKQ and physical fitness was low to moderate, there were indications that better nutrition knowledge might result in increased physical fitness and, consequently, soccer performance.

## Figures and Tables

**Table 1:** Nutrition knowledge of participants.

	Agree		Disagree		Don't know	
	No	%	No	%	No	%
Macronutrient statements						
Carbohydrate and fat are the main energy sources^a^	116	62.7	33	17.8	36	19.5
Should not eat sweets prior to an event^b^	112	60.5	51	27.6	21	11.4
Eating carbohydrates makes you fat^b^	48	25.9	94	50.8	42	22.7
Should consume high-fat meals 2 to 3 hours before an event^b^	52	28.1	107	57.8	26	14.1
Protein is the main energy source for the muscle^b^	163	88.1	7	3.8	15	8.1
Protein supplements are necessary^b^	72	38.9	72	38.9	41	22.2
Hydration statements						
Should replace fluids before, during and after an event^a^	178	96.2	4	2.2	3	1.6
Sports drinks are better than water^a^	73	39.5	75	40.5	37	20.0
Should rely on thirst to ensure fluid replacement^b^	22	11.9	122	65.9	40	21.6
Dehydration decreases performance^a^	162	87.6	8	4.3	15	8.1
Micronutrient statement						
Vitamin and mineral supplements increase energy levels^b^	136	73.5	12	6.5	37	20.0

^a^All these statements are true; ^b^all these statements are false.

**Table 2:** Anthropometry, body composition, flexibility, and aerobic and anaerobic power of soccer players by nutritional knowledge.

	Total (*n* = 185)	Low NK (*n* = 57)	Moderate NK (*n* = 78)	High NK (*n* = 50)	Comparison
Age (yr)	21.3 ± 4.9	19.7 ± 4.1^H^	20.8 ± 4.2^H^	24.1 ± 5.8^L,M^	*F*_2,182_ = 13.1, *p* < 0.001, *η* ^2^ = 0.13
Weight (kg)	72.3 ± 8.4	70.1 ± 8.6^H^	72.3 ± 8.5	74.6 ± 7.3^L^	*F*_2,182_ = 3.9, *p* = 0.021, *η* ^2^ = 0.04
Height (cm)	177.5 ± 6.4	176.4 ± 6.3	177.1 ± 6.5	179.3 ± 6.3	*F*_2,182_ = 2.8, *p* = 0.062, *η* ^2^ = 0.03
BMI (kg*·*m^−2^)	22.9 ± 2.0	22.5 ± 2.2	23.0 ± 2.1	23.2 ± 1.6	*F*_2,182_ = 1.7, *p* = 0.182, *η* ^2^ = 0.02
BF (%)	14.5 ± 3.8	14.6 ± 3.5	14.5 ± 4.1	14.4 ± 3.8	*F*_2,182_ < 0.1, *p* = 0.963, *η* ^2^ < 0.01
FM (kg)	10.7 ± 3.7	10.4 ± 3.4	10.7 ± 3.9	10.9 ± 3.6	*F*_2,182_ = 0.2, *p* = 0.780, *η* ^2^ < 0.01
FFM (kg)	61.6 ± 6.2	59.7 ± 6.3^H^	61.7 ± 6.4	63.7 ± 5.2^L^	*F*_2,182_ = 5.7, *p* = 0.004, *η* ^2^ = 0.06
SAR (cm)	24.5 ± 7.0	22.6 ± 7.0	25.2 ± 6.8	25.6 ± 7.0	*F*_2,181_ = 3.1, *p* = 0.046, *η* ^2^ = 0.03
PWC_170_ (W)	205 ± 31	201 ± 42	207 ± 47	205 ± 31	*F*_2,177_ = 0.3, *p* = 0.745, *η* ^2^ < 0.01
PWC_170_ (W*·*kg^−1^)	2.83 ± 0.48	2.86 ± 0.49	2.85 ± 0.53	2.75 ± 0.39	*F*_2,177_ = 0.8, *p* = 0.439, *η* ^2^ = 0.01
*P*_peak_ (W)	816 ± 122	782 ± 112^H^	825 ± 129	840 ± 115^L^	*F*_2,178_ = 3.4, *p* = 0.034, *η* ^2^ = 0.04
*P*_peak_ (W*·*kg^−1^)	11.3 ± 1.0	11.1 ± 0.9	11.4 ± 1.0	11.3 ± 1.0	*F*_2,178_ = 1.2, *p* = 0.313, *η* ^2^ = 0.01
*P*_mean_ (W)	618 ± 86	597 ± 88	620 ± 84	637 ± 84	*F*_2,172_ = 2.8, *p* = 0.063, *η* ^2^ = 0.03
*P*_mean_ (W*·*kg^−1^)	8.54 ± 0.82	8.51 ± 0.83	8.57 ± 0.86	8.52 ± 0.76	*F*_2,172_ = 0.1, *p* = 0.895, *η* ^2^ < 0.01
FI (%)	45.0 ± 6.9	44.2 ± 5.7	45.9 ± 7.7	44.7 ± 6.8	*F*_2,172_ = 1.0, *p* = 0.381, *η* ^2^ = 0.01

NK = nutritional knowledge, BMI = body mass index, BF = body fat, FM = fat mass, FFM = fat free mass, SAR = sit-and-reach test, PWC_170_ = physical working capacity in heart rate 170 bpm, *P*_peak_ = peak power, *P*_mean_ = mean power, and FI = fatigue index.

Letters H, L, and M when presenting as superscripts denote difference from group with high, low, and moderate nutritional knowledge, respectively.

**Table 3:** Pearson correlations of nutritional knowledge with anthropometry, body composition, flexibility, and aerobic and anaerobic power.

	Pearson *r*
Age (yr)	0.34^‡^
Weight (kg)	0.21^†^
Height (cm)	0.20^†^
BMI (kg*·*m^−2^)	0.11
BF (%)	−0.02
FM (kg)	0.05
FFM (kg)	0.25^‡^
SAR (cm)	0.15∗
PWC_170_ (W)	0.04
PWC_170_ (W*·*kg^−1^)	−0.09
*P*_peak_ (W)	0.18∗
*P*_peak_ (W*·*kg^−1^)	0.04
*P*_mean_ (W)	0.20^†^
*P*_mean_ (W*·*kg^−1^)	0.01
FI (%)	0.00

BMI = body mass index, BF = body fat, FM = fat mass, FFM = fat free mass, SAR = sit-and-reach test, PWC_170_ = physical working capacity in heart rate 170 bpm, *P*_peak_ = peak power, *P*_mean_ = mean power, and FI = fatigue index.

*P < 0.05, ^†^ *P* < 0.01 and ^‡^ *P* < 0.001.
